# Diagnostic role of fine needle aspiration cytology (FNAC) in the evaluation of salivary gland swelling: an institutional experience

**DOI:** 10.1186/s13104-015-1048-5

**Published:** 2015-03-27

**Authors:** Samreen Naz, Atif Ali Hashmi, Amna khurshid, Naveen Faridi, Muhammad Muzzammil Edhi, Anwar Kamal, Mehmood Khan

**Affiliations:** Department of Histopathology, Liaquat National Hospital and Medical College, Karachi, Pakistan; Liaquat National Hospital and Medical College, Karachi, Pakistan; Dhaka Medical College, Dhaka, Bangladesh

**Keywords:** Salivary gland, Fine needle aspiration (FNAC), Parotid gland, Submandibular gland

## Abstract

**Background:**

Fine needle aspiration cytology (FNAC) is a cytodiagnostic method based on morphologic findings of individual and small group of cells aspirated using a fine needle. The aim of the present study is to evaluate the spectrum of salivary gland lesions in our setting and to assess the diagnostic accuracy of FNAC for salivary gland lesions.

**Methods:**

The study involved 187 cases of parotid and submandibular swellings of patients who underwent FNAC at our institution. Thirty one (31) patients with a FNAC diagnosis of neoplastic lesion subsequently underwent excision biopsies. The results of FNAC and final histology were compared and accuracy of FNAC was determined.

**Results:**

Mean age of patients was 42 (±21) years and male to female ratio was 1:1. Chronic sialadenitis was the most common non-neoplastic lesion (33.8%) followed by acute and chronic sialadenitis (29.7%) and chronic granulomatous inflammation (27.0%). Pleomorphic adenoma was the most common benign neoplasm and non-Hodgkin’s lymphoma was the most common malignant lesion (38.9%) followed by acinic cell (27.8%) and adenoid cystic carcinoma (16.7%). Total 31 patients subsequently underwent surgical excision, out of which 21 were benign and 9 were malignant, 20 cases (64.5%) were of pleomorphic adenoma, 3 cases (9.6%) of acinic cell carcinoma, 2 cases (6.4%) each of warthin tumor, adenoid cystic carcinoma and non-hodgkin lymphoma and 1 case (3.2%) each of mucoepidermoid carcinoma and mucinous adenocarcinoma. The overall accuracy of FNAC in our study was found to be 83.8% with 77.7% sensitivity and 86.3%, specificity. The revised sensitivity and specificity after adjusting verification bias were 68.5% and 91% respectively. False negative diagnosis was rendered in mucoepidermoid carcinoma and acinic cell carcinoma whereas false positive diagnosis was given in cases of pleomorphic adenoma.

**Conclusion:**

We found a good concordance between FNAC and histology, however pleomorphic adenoma may impart a diagnostic challenge when inadequately aspirated and therefore we advice either immunohistochemical studies (if cell block material is available) or repeat aspiration in difficult cases.

## Background

Fine needle aspiration cytology (FNAC) is a cytodiagnostic method based on morphologic findings of individual and small group of cells aspirated using a fine needle. FNAC was introduced in 1920’s and soon it gained wide acceptance among clinicians due to ease of its performance and rapidity of diagnosis [1]. Nowadays FNAC has become a cornerstone diagnostic tool in head and neck swellings [2,3].

The role of FNAC in suspected salivary gland swellings is two folds. Firstly to confirm the origin as preauricular and submandibular lymph node swellings can mimic salivary gland neoplasm clinically and secondly to get a preliminary diagnosis about the nature of the disease process before embarking on definite management plan. FNAC is a reliable method to differentiate between inflammatory and neoplastic lesions. FNAC diagnosis of neoplastic process even when benign usually lead to surgical excision. Although diagnostic accuracy of FNAC in the assessment of salivary gland swellings has been studied in various studies, it has not been widely assessed in our set up [4-7]. The aim of the present study is to evaluate the spectrum of salivary gland lesions in our setting and to assess the diagnostic accuracy of FNAC for salivary gland lesions.

## Methods

This is a retrospective observational study performed in the department of histopathology, Liaquat National Hospital for duration of four (4) years. The study involved 187 cases who presented with parotid and submandibular swellings. The study was approved by Liaquat National hospital and medical college research and ethical review committee. FNAC was performed using a 22 – 23 gauge needle attached to a disposable syringe with plunger under aseptic conditions. Smears were performed and slides were stained with haemotoxylin and eosin, Papanicolou and giemsa methods. Remaining material was processed as cell block preparation and processed according to standard guidelines. Thirty one (31) patients with a FNAC diagnosis of neoplastic lesion subsequently underwent excision biopsies. After gross examination of specimens, the representative sections were processed and examined by H & E methods. Special and immunohistochemical stains were performed when necessary. The results of FNAC and final histology were compared and accuracy of FNAC was determined.

### Statistical analysis

The cytological and histological analysis was reported in terms of frequencies and percentages. Furthermore, diagnostic accuracy of FNAC for salivary gland swellings was measured using Histopathology as Gold Standard. Sensitivity, Specificity, NPV and PPV were calculated using formulas listed below.

$$ \mathrm{P}\mathrm{P}\mathrm{V}=\frac{\mathrm{number}\ \mathrm{of}\ \mathrm{true}\ \mathrm{positives}}{\mathrm{number}\ \mathrm{of}\ \mathrm{true}\ \mathrm{positives} + \mathrm{number}\ \mathrm{of}\ \mathrm{false}\ \mathrm{positives}}=\frac{\mathrm{number}\ \mathrm{of}\ \mathrm{true}\ \mathrm{positives}}{\mathrm{number}\ \mathrm{positives}\ \mathrm{calls}} $$

$$ \mathrm{N}\mathrm{P}\mathrm{V}=\frac{\mathrm{number}\ \mathrm{of}\ \mathrm{true}\ \mathrm{negative}\mathrm{s}}{\mathrm{number}\ \mathrm{of}\ \mathrm{true}\ \mathrm{negative}\mathrm{s} + \mathrm{number}\ \mathrm{of}\ \mathrm{false}\ \mathrm{negative}\mathrm{s}}=\frac{\mathrm{number}\ \mathrm{of}\ \mathrm{true}\ \mathrm{negative}\mathrm{s}}{\mathrm{number}\ \mathrm{negative}\ \mathrm{calls}} $$

$$ \mathrm{accuracy} = \frac{\mathrm{number}\ \mathrm{of}\ \mathrm{true}\ \mathrm{positives} + \mathrm{number}\ \mathrm{of}\ \mathrm{true}\ \mathrm{negatives}}{\mathrm{number}\ \mathrm{of}\ \mathrm{true}\ \mathrm{positives} + \kern0.5em \mathrm{false}\ \mathrm{positives} + \mathrm{false}\ \mathrm{negatives} + \mathrm{true}\ \mathrm{negatives}} $$

$$ \mathrm{sensitivity} = \frac{\mathrm{number}\ \mathrm{of}\ \mathrm{true}\ \mathrm{positives}}{\mathrm{number}\ \mathrm{of}\ \mathrm{true}\ \mathrm{positives} + \mathrm{number}\ \mathrm{of}\ \mathrm{false}\ \mathrm{negatives}} $$

$$ \mathrm{specificity} = \frac{\mathrm{number}\ \mathrm{of}\ \mathrm{true}\ \mathrm{negatives}}{\mathrm{number}\ \mathrm{of}\ \mathrm{true}\ \mathrm{negatives} + \mathrm{number}\ \mathrm{of}\ \mathrm{false}\ \mathrm{positives}} $$

In addition, Sensitivity and Specificity were also adjusted for verification bias.

$$ \mathrm{Sens} = \left(\mathrm{T} = 1\ /\ \mathrm{D} = 1\right) = \frac{\frac{{\mathrm{n}}_1{\mathrm{s}}_1}{{\mathrm{s}}_1 + {\mathrm{r}}_1}}{\frac{{\mathrm{n}}_1{\mathrm{s}}_1}{{\mathrm{s}}_1 + {\mathrm{r}}_1}+\frac{{\mathrm{n}}_2{\mathrm{s}}_2}{{\mathrm{s}}_2 + {\mathrm{r}}_2}} $$

$$ \mathrm{S}\mathrm{p}\mathrm{e} = \mathrm{p}\left(\mathrm{T} = 0\ /\ \mathrm{D} = 0\right) = \frac{\frac{{\mathrm{n}}_2{\mathrm{s}}_2}{{\mathrm{s}}_2 + {\mathrm{r}}_2}}{\frac{{\mathrm{n}}_1{\mathrm{s}}_1}{{\mathrm{s}}_1 + {\mathrm{r}}_1}+\frac{{\mathrm{n}}_2{\mathrm{s}}_2}{{\mathrm{s}}_2 + {\mathrm{r}}_2}} $$

## Results

The mean age of patients was 42 (±21) years and male to female ratio was 1:1. Out of 187 patients who underwent FNAC, 8 cases were non-diagnostic due to lack of adequate material, 74 were non-neoplastic/inflammatory. In 49 cases a diagnosis of neoplastic process was rendered, out of which 23 were benign and an unequivocal diagnosis of malignancy was given in 18 (9.6%) cases as shown in Table 1. Chronic sialadenitis was the most common non-neoplastic lesion (33.8%) followed by acute and chronic sialadenitis (29.7%) and chronic granulomatous inflammation (27.0%) (Table 2). Pleomorphic adenoma was the most common benign neoplasm (Table 3) and non-Hodgkin’s lymphoma was the most common malignant lesion (38.9%) followed by acinic cell (27.8%) and adenoid cystic carcinoma (16.7%) (Table 4).

**Table 1:** **Cytologic diagnostic categories**

**Cytologic diagnostic category**	**Frequency (n)**	**Percentage (%)**
Non-diagnostic	8	4.3%
Inflammatory/non-neoplastic	74	39.6%
Benign neoplasm	64	34.2%
Suspicious for malignancy	23	12.2%
Malignant	18	9.6%

**Table 2:** **Cytologic diagnosis of non-neoplastic lesions**

**Cytological diagnosis**	**Frequency (n)**	**Percentage (%)**
Chronic granulomatous inflammation	20	27.0%
Chronic sialadenitis	25	33.7%
Acute on chronic sialdenitis	22	29.7%
Benign cystic lesion	7	9.4%
Total	74	39.5%

**Table 3:** **Cytologic diagnosis of benign neoplasms**

**Cytological diagnosis**	**Frequency (n)**	**Percentage (%)**
Pleomorphic adenoma	57	89.0%
Warthin tumor	7	10.9%
Total	64	34.2%

**Table 4:** **Cytologic diagnosis of malignant neoplasms**

**Cytological diagnosis**	**Frequency (n)**	**Percentage (%)**
Mucoepidermoid carcinoma	2	11.1%
Acinic cell carcinoma	5	27.7%
Adenoid cystic carcinoma	3	16.6%
Lymphoproliferative disorder	7	38.8%
Papillary adenocarcinoma	1	5.5%
Total	18	9.6%

Out of these 187 cases, 31 patients subsequently underwent surgical excision/histologic evaluation, out of which 21 were benign and 9 were malignant, Total 31 patients subsequently underwent surgical excision, out of which 21 were benign and 9 were malignant, 20 cases (64.5%) were of pleomorphic adenoma, 3 cases (9.6%) of acinic cell carcinoma, 2 cases (6.4%) each of warthin tumor, adenoid cystic carcinoma and non-hodgkin lymphoma and 1 case (3.2%) each of mucoepidermoid carcinoma and mucinous adenocarcinoma. Table 5 shows the comparison of cytologic and histologic diagnosis. Overall accuracy of FNAC in our study was found to be 83.8% with 77.7% sensitivity and 86.3%, specificity as shown in Table 6. The revised sensitivity and specificity after adjusting verification bias were 68.5% (37.9-99.2) and 91% (82.7-99.3) respectively (Table 7).

**Table 5:** **Comparison of cytologic and histologic diagnosis**

**Cytologic diagnosis**	**Histologic diagnosis**							
		**Pleomorphic adenoid**	**Mucoepidermoid carcinoma**	**Mucinous adenocarcinoma**	**Adenoid cystic carcinoma**	**Nonhodgkin lymphoma**	**Acinic cell carcinoma**	**Warthin tumor**
	**Total**	**Frequency (n)**	**Frequency (n)**	**Frequency (n)**	**Frequency (n)**	**Frequency (n)**	**Frequency (n)**	**Frequency (n)**
Pleomorphic adenoma	19	17	1		1			
Warthin tumor	2							2
Suspicious for malignancy	2	1		1				
Mucoepidermoid carcinoma	1	1						
Acinic cell carcinoma	3						3	
Adenoid cystic carcinoma	2	1			1			
Lymphoproliferative disorder	2					2

**Table 6:** **Diagnostic accuracy of FNAC for Salivary gland swelling**

Sensitivity	77.7%
Specificity	86.3%
Positive predictive value	70%
Negative predictive value	90.4%
Accuracy	83.3%

**Table 7:** **Revised sensitivity and specificity after adjusting verification bias**

		**95% confidence interval**	
		**Upper limit**	**Lower limit**
Sensitivity	68.5%	37.9%	99.2%
Specificity	91.0%	82.7%	99.3%

## Discussion

Swelling of salivary glands, specifically parotid and submandibular gland presents as a common problem and being readily visible creates havoc among patients. In addition parotid/submandibular swellings also remain a diagnostic challenge among clinicians. FNAC provides a convenient way to obtain a tissue based diagnosis and therefore has now become a diagnostic test of choice to solve this dilemma. Our study explains the role of this procedure in our setup to diagnose salivary gland lesions and the spectrum of disease pathology in our population.

Literature review revealed a wide variation in the sensitivity and specificity of FNAC for salivary gland swelling in different populations and setups [8-10]. Zerpa et al. studied 93 cases of parotid gland tumors, revealing a sensitivity and specificity of 57% and 95% respectively [11]. On the other hand, Pastore et al. found a sensitivity and specificity of 83% and 93% respectively. They evaluated 357 cases of salivary gland lesions [12]. Similarly Jaein et al. revealed 92.8% sensitivity and 93.9% specificity in a study involving 80 cases of salivary gland swellings, out of which 14 cases were of malignant salivary gland neoplasms [13]. Kim et al. found a diagnostic accuracy of FNAC to be 92% in differentiating malignant from benign salivary gland tumors [14]. Fakhry et al. evaluated 249 parotid tumors, out of which 75% were benign and 25% were malignant. The sensitivity and specificity to detect malignancy was assessed to be 80% and 89.5% respectively. They found 16 false positive results, among which warthin’s tumor and pleomorphic adenoma were most common, while false negative diagnosis were given in cases of lymphomas and mucoepidermoid carcinomas. The diagnostic accuracy for benign and malignant tumors was 16% and 44% respectively [15].

We found an overall diagnostic accuracy of FNAC to be 83.8%. There were two cases of false negative diagnosis. These two cases were one each of mucoepidermoid carcinoma and acinic cell carcinoma which were initially diagnosed as pleomorphic adenoma on FNAC. Pleomorphic adenoma is a biphasic neoplasm. The epithelial component may present a variety of histologic patterns including squamous and ductal structures and they may sometimes exhibit significant cytologic atypia. Lack of stromal component in the aspirated material may lead to a false positive diagnosis especially that of low grade mucoepidermoid carcinoma which may show diversity in the morphologic patterns of epithelial components including squamous, intermediate and mucinous cells. There were 3 cases of false positive diagnosis. In one of that case there were extensive squamous elements without any other component and therefore a diagnosis of neoplastic lesion, suspicious for malignancy was given with a possibility of metastatic squamous cell carcinoma and mucoepidermoid carcinoma. The final histology revealed the diagnosis of pleomorphic adenoma. The other two cases with false negative diagnosis were that of mucoepidermoid and adenoid cystic carcinoma which were inaccurately diagnosed on FNAC as pleomorphic adenoma. Adenoid cystic carcinoma is a close differential of pleomorphic adenoma and basal cell adenoma. This differentiation is very important as the surgical management is different. Adenoid cystic carcinoma shows basement membrane like material which may be misinterpreted as stromal component. The epithelial component of adenoid cystic carcinoma is usually very bland leading to inaccurate impression of benignancy. Immunohistochemical studies on cell block material may be very helpful in this distinction including stains for basal lamina and CD117 stain which is positive in adenoid cystic carcinoma.

A few studies were also conducted in Pakistan, evaluating the role of FNAC in salivary gland pathology. A study conducted in Pakistan including 129 cases of parotid gland lesions, revealed 98 benign and 31 malignant neoplasms. Pleomorphic adenoma was the most common benign tumor while mucoepidermoid carcinoma being the most common malignant diagnosis [16]. In our study, although pleomorphic adenoma was the most common benign tumor, however the most common malignant tumor was acinic cell carcinoma.

## Conclusion

In conclusion, we found a good concordance between FNAC and final histology, however pleomorphic adenoma may impart a diagnostic challenge when inadequately aspirated and therefore either immunohistochemical studies should be done if cell block material is available or repeat aspiration should be advised before embarking on therapeutic excision.

## Abbreviations

FNAC: Fine needle aspiration cytology
H& E: Hematoxylin and eosin
